# Biological Functions and Health Benefits of Eggshell Calcium: From Bone Health to Cardiovascular Risk Factors: A Comprehensive Review

**DOI:** 10.3390/foods15213790

**Published:** 2026-10-23

**Authors:** Vu Thi Thu Hien, Thi Anh Tho Pham, Lai Thi Cam Nhung, Nhat Linh Nguyen, Yumi Takeda, Naoki Fujiwara, Mai Thuy Pham, Ryosuke Matsuoka, Shigeru Yamamoto, Michihiro Sugano

**Affiliations:** 1 National Institute of Nutrition, Hanoi 120000, Vietnam; hienvuthithu.nin@gmail.com (V.T.T.H.); nhatlinhnnl22012005@gmail.com (N.L.N.); 2 Department of Nutrition, Aomori University of Health and Welfare, Aomori 030-8505, Japan; anhtho@kewpie.com.vn (T.A.T.P.); yamamotoshigeru426@gmail.com (S.Y.); 3 Kewpie Vietnam Co., Ltd., Hanoi 120000, Vietnam; camnhung081101@gmail.com; 4 R&D Division, Kewpie Corporation, Tokyo 182-0002, Japan; yumi_takeda@kewpie.co.jp (Y.T.); naoki_fujiwara@kewpie.co.jp (N.F.); 5 Asian Nutrition and Food Culture Research Center, Jumonji University, Niiza 352-8510, Japan; maipth25@gmail.com; 6 Kyushu University, Fukuoka 819-0395, Japan; suganomi@deluxe.ocn.ne.jp; 7 Prefectural University of Kumamoto, Kumamoto 862-8502, Japan; 8 Special Adviser of the Japan Egg Science Society, Tokyo 182-0002, Japan

**Keywords:** eggshell, calcium, eggshell membrane, calcium absorption, bone mineral density, blood pressure, fat absorption

## Abstract

A hen’s egg consists of an eggshell, eggshell membrane, egg yolk, and egg white, among which the egg yolk and egg white are consumed. Eggshells are usually discarded. The term eggshell is used to refer to the eggshell including the eggshell membrane, and both the eggshell membrane and the eggshell after removal of the eggshell membrane are known to exhibit health-promoting functions. The major component of the eggshell membrane is protein, and the major component of the eggshell after removal of the eggshell membrane is calcium (calcium carbonate). A characteristic feature of eggshells is their porous structure, and they are known to dissolve readily in gastric acid upon ingestion. Because dissolution by gastric acid is essential for absorption of dietary calcium, eggshells have attracted attention as a calcium source with high digestibility and bioavailability. High phosphorus intake inhibits calcium absorption, but eggshell calcium has low phosphorus content. This review discusses eggshell calcium’s health benefits: contributing to bone health (SOS in bone +40 m/s from baseline) and reduced blood pressure (systolic −8 mmHg, diastolic −4 mmHg from baseline). It also covers eggshell membrane’s absorbability, knee pain relief, and skin hydration improvement.

## 1. Introduction

Chicken eggs contain all nutrients except dietary fiber and vitamin C and are regarded as a highly nutritious food [1]. However, because eggs also contain cholesterol, The Japan Atherosclerosis Society’s Guidelines for the Prevention of Atherosclerotic Diseases (2022) list eggs as a food that should be limited in the dietary management of high LDL-cholesterol [2]. We previously published a review on eggs and cholesterol and demonstrated that eggs can be safely consumed [3]. In addition, because egg white consists primarily of protein on a dry-matter basis, we have investigated the health-promoting functions of egg white and reported that egg white protein exerts cholesterol-lowering and visceral fat-reducing effects [4,5,6,7]. Choline, a component unique to egg yolk, is a precursor to the neurotransmitter acetylcholine and was therefore expected to improve cognitive function. An improvement in cognitive function resulting from the combined administration of egg yolk choline and vitamin B_12_, which serves as an acetyl group donor, has been reported [8]. In recent years, clinical trials in humans have reported that egg yolk choline improves verbal memory [9,10]. Consumption of hen’s eggs has been reported to reduce the risk of dementia [11,12], and this protective effect has been suggested to be attributable to choline in egg yolk. Thus, various health-promoting effects have been reported for the edible parts of egg (egg white and egg yolk).

In contrast, when eggs are consumed, the egg yolk and egg white are utilized as edible components, whereas eggshells have rarely been used as food materials.

Therefore, from the perspective of effective utilization of unused resources, the use of eggshells represents an important approach toward achieving the Sustainable Development Goals.

The term eggshell includes both the mineralized matrix (mineralized shell) and the shell membranes (eggshell membranes), and the separate utilization of the eggshell membrane and the eggshell after removal of the membrane is being promoted. In the past, eggshells after removal of the eggshell membrane have mainly been used in non-food applications, such as fertilizers, de-icing agents, and chalk, whereas eggshell membranes have been used as raw materials for cosmetics, tissue paper, and formaldehyde adsorbents in housing materials.

The major component of the eggshell membrane is protein, whereas the major component of the eggshell after removal of the eggshell membrane is calcium, primarily in the form of calcium carbonate.

Regarding calcium derived from unused resources such as eggshells, calcium obtained from coral, oyster shell electrolytes, scallop shells, and lobster shells has been reported to suppress reductions in bone mineral density in ovariectomized rats [13,14,15,16].

Although reviews on eggshell calcium have been published to date, descriptions of the eggshell have been limited, and these reviews also include discussions of the functions of common calcium and polyphenols. Therefore, no review focusing specifically on eggshell calcium has been published to date [17]. A literature search was conducted to determine whether any review articles have been published regarding the effects of eggshell calcium on bone. A search on PubMed using the term “eggshell calcium” yielded 50 results, while the combination of “eggshell calcium” and “review” yielded 5 results. Upon closer examination of these 5 papers, no review article specifically focused on eggshell calcium was identified. In Scopus, a search for “eggshell calcium” yielded 29 results, while the combination of “eggshell calcium” and “review” yielded 0 results (search date: 16 October 2026). This review provides an overview of the health-promoting functions of eggshell calcium and eggshell membrane, focusing specifically on these components. This paper is the first comprehensive review dedicated specifically to eggshell calcium. It is novel in that it systematically organizes the health-related functions of eggshell calcium in isolation, distinguishing itself from existing reviews that cover broader topics—such as general calcium or the combined effects of calcium and polyphenols.

## 2. Eggshell Calcium

**Figure 1:**
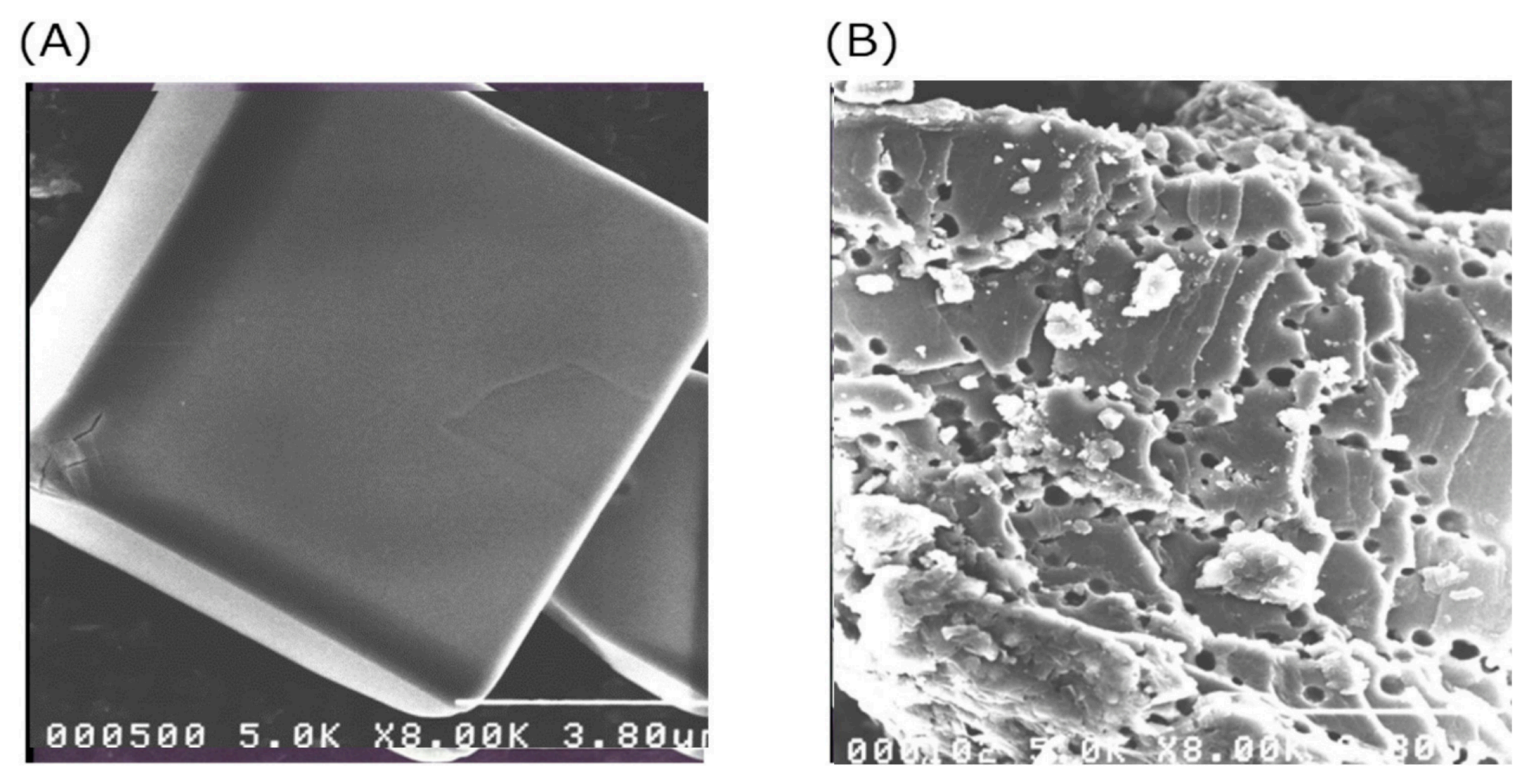
Microscopic photographs of eggshell calcium and calcium carbonate (8000×). (**A**) Calcium carbonate; (**B**) eggshell calcium.

The composition of the eggshell is shown in Table 1. Eggshell calcium is primarily composed of calcium carbonate and contains 37.9 g of calcium per 100 g. In addition, eggshell calcium is characterized by its low phosphorus content, because phosphorus inhibits calcium absorption. A micrograph of eggshell calcium at an 8000× magnification is shown in Figure 1. Whereas reagent-grade calcium carbonate exhibits a crystalline structure, eggshell calcium exhibits a porous structure. Due to this porous structure, eggshell calcium has a large surface area available for contact with gastric acid after ingestion. Because calcium carbonate absorption is reduced when gastric acid secretion is insufficient [18], dissolution of calcium in gastric acid is essential for calcium absorption. Eggshell calcium is known as a highly bioavailable calcium source. The details are described below.

In a study evaluating the metabolism of eggshell calcium with and without vitamin D supplementation, ovariectomized rats fed a vitamin D-supplemented diet showed significant differences in intestinal calcium absorption, urinary calcium excretion, and fecal calcium excretion compared with rats fed a diet without vitamin D supplementation. Because its effects were equivalent to those of calcium carbonate, eggshell calcium was confirmed to function as a calcium source [19].

## 3. Safety of Eggshell Calcium

The Ministry of Health, Labour and Welfare, Japan, lists eggshell calcium as a food additive (fortifying agent) [20], and products fortified with eggshell calcium have been marketed for many years, indicating sufficient dietary experience.

In safety studies, no abnormalities were observed in rats following continuous high-dose administration of 1000 mg/kg/day for 91 days [21]. A reverse mutation assay was performed using bacteria (*Escherichia coli* strain WP2 *uvr*A and four *Salmonella* Typhimurium TA strains), and negative results were obtained for all strains [22]. Because eggshell calcium is derived from chicken eggs, concerns remained regarding the potential persistence of allergenicity. Therefore, in an in vitro enzyme-linked immunosorbent assay (ELISA) and an in vivo Passive Cutaneous Anaphylaxis (PCA) test, the antigenicity of eggshell calcium against egg white proteins was confirmed to be extremely low [23]. In addition, while diagnostic methods for food allergies such as blood tests and skin tests are available, it is known that some individuals can still consume the food even with a positive result on these tests [24], and an accurate allergy diagnosis is required to improve the quality of life (QOL) of such people. One diagnostic method developed in this context is the oral food challenge. Therefore, we conducted an oral food challenge using eggshell calcium on six children aged 3 to 6 years with egg allergies. The subjects ingested 1 g of eggshell calcium in small amounts over the course of one hour and were observed for 24 hours; no cutaneous, gastrointestinal, or respiratory symptoms were observed [25]. Although eggshells are known to contain small amounts of protein [25,26], the possibility of cross-reactivity between eggshell proteins and egg white proteins was considered low.

In 45 pre- and postmenopausal women who consumed a supplement containing eggshell calcium providing 300 mg/day of calcium for 12 weeks, no adverse events attributable to long-term intake of the product were observed, and hematology test results, as well as liver function, renal function, blood mineral concentrations, blood lipids, blood glucose levels, and urinalysis findings, were within the range of physiological variation [27].

When 15 healthy adults consumed eggshell calcium providing 1250 mg of calcium per day for four weeks, no adverse events attributable to the long-term intake of the product occurred; furthermore, results from hematological tests, liver and renal function tests, blood mineral concentrations, blood lipids, blood glucose levels, and urinalysis all fell within the range of physiological variation [28].

Eggshell calcium is characterized by its low phosphorus content, making it an advantageous choice of calcium source for individuals with impaired renal function or those who need to manage their dietary phosphorus intake.

Adverse effects associated with excessive calcium intake include hypercalcemia, hypercalciuria, soft tissue calcification, urinary tract stones, impaired absorption of iron and zinc, and constipation [29]. Based on these findings, the Tolerable Upper Intake Level for calcium in Japanese adults is set at 2500 mg/day [30]. While it is unlikely that one would exceed this amount through a normal diet, caution is advised, as it is possible to surpass this level when taking supplements.

## 4. Solubility and Bioavailability of Eggshell Calcium

**Figure 2:**
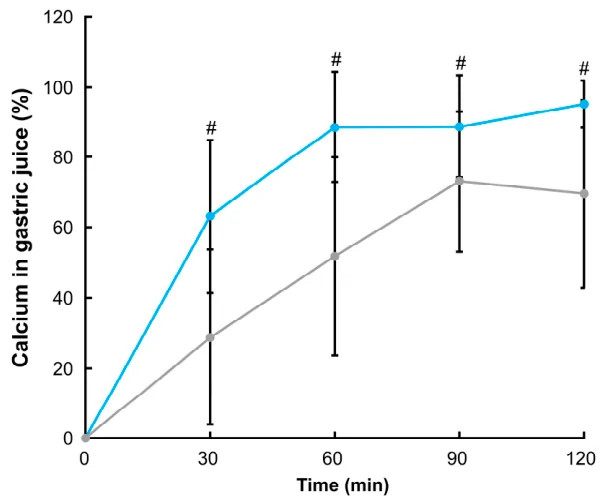
Solubility of eggshell calcium in gastric acid; blue: eggshell calcium; gray: calcium carbonate. Mean ± SEM. #: *p* < 0.05 vs. calcium carbonate [31].

There are various mechanisms involved in the digestion and absorption of calcium; one such mechanism reported is that it dissolves in gastric acid before being absorbed [18]. The following study compared the solubility of eggshell calcium in gastric acid with that of calcium carbonate. Nineteen elderly individuals receiving enteral nutrition through a gastrostomy or gastric tube were divided into two groups and administered either eggshell calcium or calcium carbonate. Gastric fluid was collected every 20 min to determine the calcium dissolution rate. Eggshell calcium exhibited a higher dissolution rate in gastric fluid than calcium carbonate (Figure 2) [31]. Although this study was conducted on elderly subjects, and the possibility that gastric acid secretion differs from that of healthy individuals cannot be ruled out, it was speculated that the following reaction occurs in the stomach:

CaCO_3_ (s) + 2H^+^ (aq) ⇌ Ca^2+^ (aq) + H_2_O (l) + CO_2_ (g).

**Figure 3:**
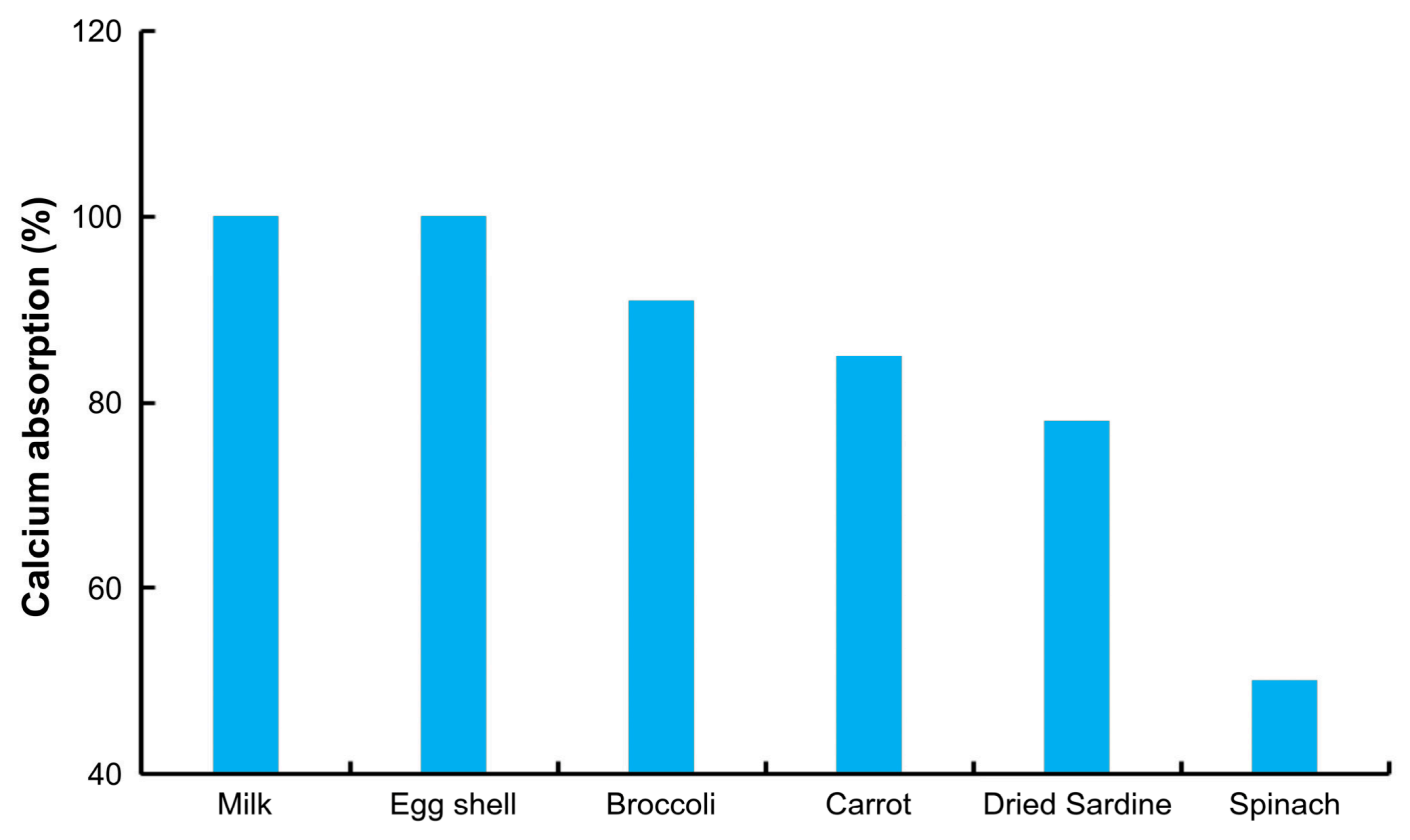
Calcium absorption rate of various foods when the calcium absorption rate of milk is 100% (the figure was made by extracting content from the table in Reference [33]).

When calcium carbonate or eggshell calcium was administered to young and mature rats, and calcium absorption was evaluated by calcium balance, eggshell calcium was found to have a higher absorption rate than calcium carbonate in mature rats [32]. Although this study was conducted more than 30 years ago, no new evidence has been reported since then; thus, this was considered a limitation. A comparison of animal studies evaluating the absorption of various calcium sources showed that the absorption rate of eggshell calcium was comparable to that of milk-derived calcium and higher than those of other calcium sources (Figure 3) [33].

Eggshells contain small amounts of protein, and studies using Caco-2 cells have demonstrated that eggshell proteins promote calcium absorption [34].

Based on these findings—specifically that the porous structure of eggshell calcium facilitates dissolution in gastric fluid, that animal studies indicate it is a calcium source with a high absorption rate, and that cell-based studies show eggshell proteins promote calcium absorption—it is suggested that eggshell calcium is a source that can be efficiently absorbed.

## 5. Effect of Eggshell Calcium on Bone Mineral Density

Individuals with a high calcium intake have been reported to have a lower risk of fractures than those with a low calcium intake [35]. This association is particularly evident in older adults and is thought to be attributable to differences in bone mineral density. Based on the results of the calcium balance study described above, eggshell calcium was shown to be a highly bioavailable calcium source. Because this suggests greater calcium retention in the body, eggshell calcium was considered to have the potential to increase bone mineral density. Therefore, the available findings regarding the effects of eggshell calcium intake on bone mineral density are summarized below.

**Figure 4:**
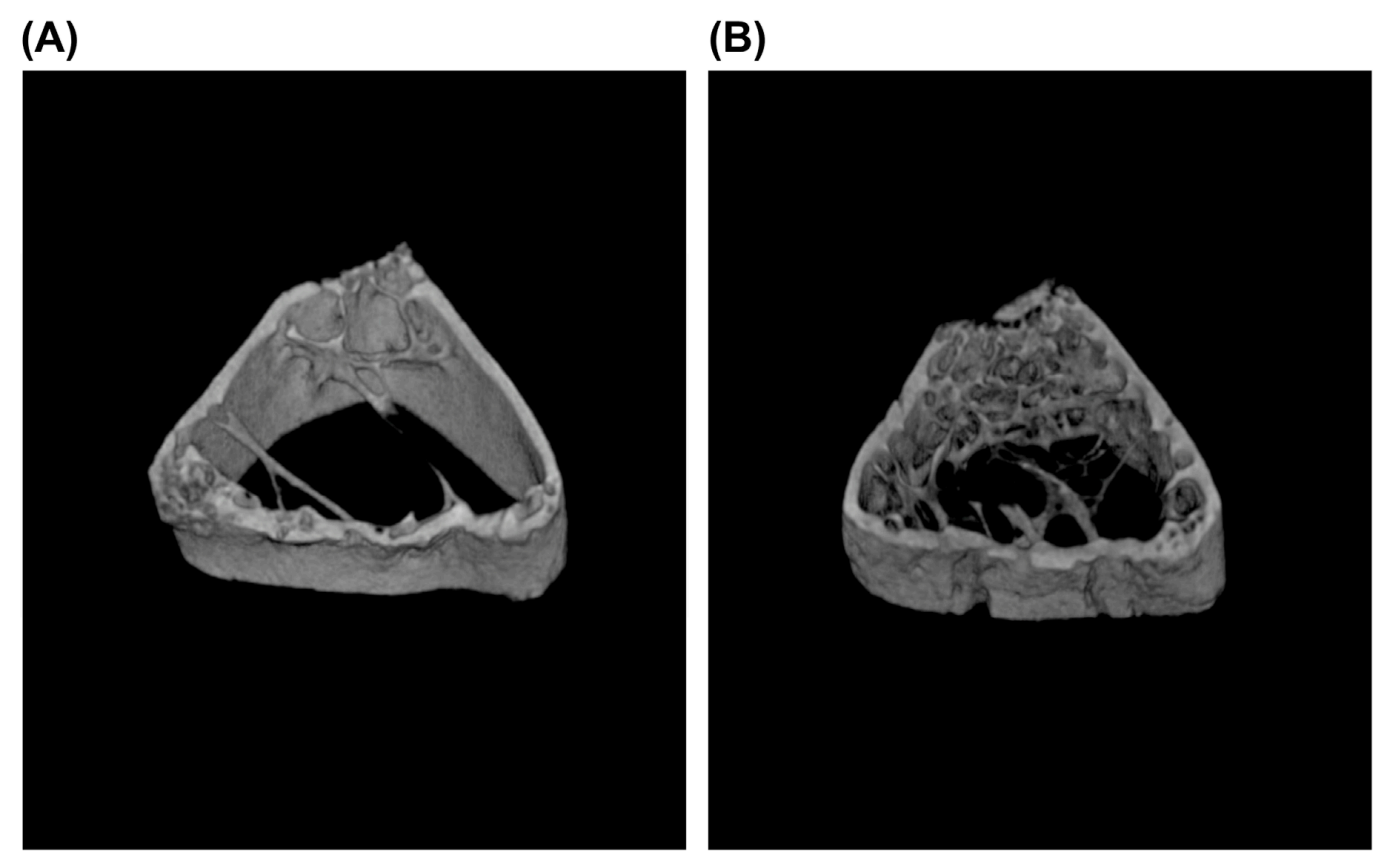
Ovariectomized mice were fed various calcium-fortified diets (calcium 0.2%) for 3 months, and their femurs were three-dimensionally imaged. (**A**) The mice fed a diet containing calcium carbonate; (**B**) the mice fed a diet containing eggshell calcium [37].

In ovariectomized rats fed diets containing either eggshell calcium or calcium carbonate for 57 days, bone mineral density of the lumbar vertebrae and proximal tibia was significantly higher in the eggshell calcium group than in the calcium carbonate group [36]. In ovariectomized mice fed diets containing either calcium carbonate or eggshell calcium for 3 months, three-dimensional imaging of the femur demonstrated that eggshell calcium suppressed the loss of bone mineral density and trabecular bone compared with calcium carbonate (Figure 4) [37].

Shizuka reported that, in rats, the absorption rate of eggshell calcium was higher than that of calcium carbonate and that eggshell calcium supplementation resulted in greater increases in femoral fracture strength and femoral calcium content than calcium carbonate supplementation [38].

Kimura et al. fed ovariectomized rats that had received a calcium-deficient diet for 30 days with diets supplemented with eggshell calcium, shell calcium, or bovine bone powder for an additional 60 days, after which cancellous bone mineral density was measured. As a result, cancellous bone mineral density was significantly increased compared with baseline in the eggshell calcium group. No significant differences were observed for the other calcium sources compared with the control group [39].

In ovariectomized rats treated with eggshell calcium or calcium carbonate for 8 weeks, eggshell calcium significantly reduced bone resorption and increased plasma calcium concentration, relative cancellous bone volume, and secondary osteon density compared with calcium carbonate, thereby contributing to improved bone strength [37].

**Figure 5:**
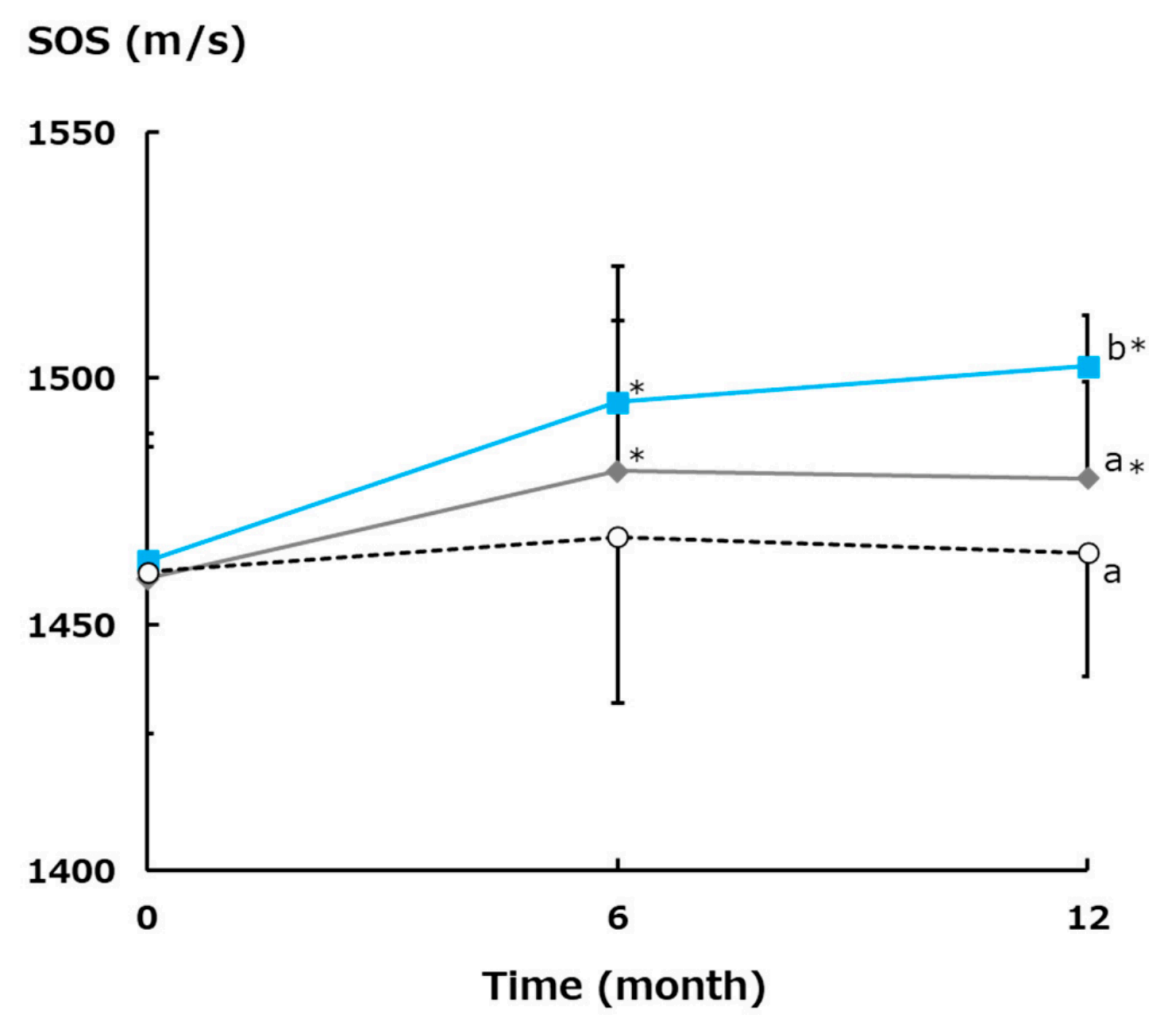
The analysis results of the SOS after ingestion of capsules. Mean ± SEM. Blue: Eggshell calcium; Gray: Calcium carbonate; Dotted line: No calcium. *: *p* < 0.05 vs. 0 month; ab: Different letters indicate a significant difference (*p* < 0.05) (this figure was modified from reference [40]).

The effects of eggshell calcium on speed of sound (SOS) in bone have also been evaluated in human studies. A single-blind, randomized, placebo-controlled, parallel-group study was conducted in Vietnamese women who were at least five years postmenopausal (mean age, 61.7 years). Participants were assigned to receive eggshell calcium containing 300 mg of calcium/day, calcium carbonate containing 300 mg of calcium/day, or placebo for 12 months. Within-group comparisons showed significant increases in SOS via Quantitative Ultrasound (QUS) at both 6 and 12 months compared with baseline in the eggshell calcium and calcium carbonate groups. Between-group comparisons of SOS at 6 and 12 months showed that, at 12 months, SOS value was significantly higher in the eggshell calcium group than in both the placebo and calcium carbonate groups [40]. These findings indicate that eggshell calcium may be effective in maintaining bone health (Figure 5).

**Figure 6:**
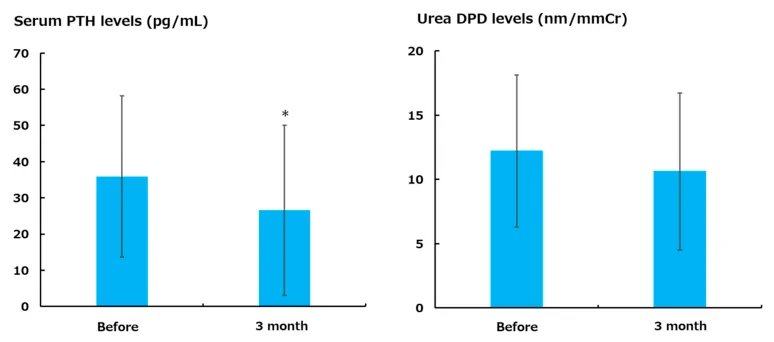
Effect of a 3-month intake of eggshell calcium (approximately 800 mg as calcium) on serum PTH and urinary DPD levels in elderly women. Mean ± SEM. *: *p* < 0.05 vs. before intake (the figure was made by extracting content from the table in Reference [41]).

When elderly women were administered 800 mg/day of eggshell calcium for 3 months, their blood parathyroid hormone concentration (PTH), which is one of the bone metabolic markers, showed a significantly lower value compared with baseline. Although no significant change was observed in urinary deoxy-pyridinoline (DPD) concentration, the mean urinary DPD concentration decreased by 14% following eggshell calcium intake compared with baseline (Figure 6) [41].

Taken together, these findings demonstrate that eggshell calcium contribute to maintaining bone health, possibly owing in part to its high bioavailability and its potential to suppress bone resorption.

## 6. Blood Pressure-Lowering Effect of Eggshell Calcium

It has been known for more than half a century that the incidence of hypertension is lower in regions with high calcium concentrations in drinking water than in regions with low calcium concentrations [42,43].

In 1999, Griffith et al. conducted a meta-analysis of 42 intervention studies on calcium intake and blood pressure, using various calcium sources but excluding eggshell calcium. Eight studies lasted at least six months, while the others lasted 4–14 weeks. Daily calcium intake exceeded 1500 mg in 17 studies, ranged from 1000 to 1500 mg in 17, and was below 1000 mg in four. No study used eggshell calcium. [44].

Because eggshell calcium is considered one of the most highly bioavailable calcium sources, we hypothesized that it would exert antihypertensive effects even at relatively low doses. Therefore, this study investigated the effects of eggshell calcium providing 600 mg/day of calcium on blood pressure.

**Figure 7:**
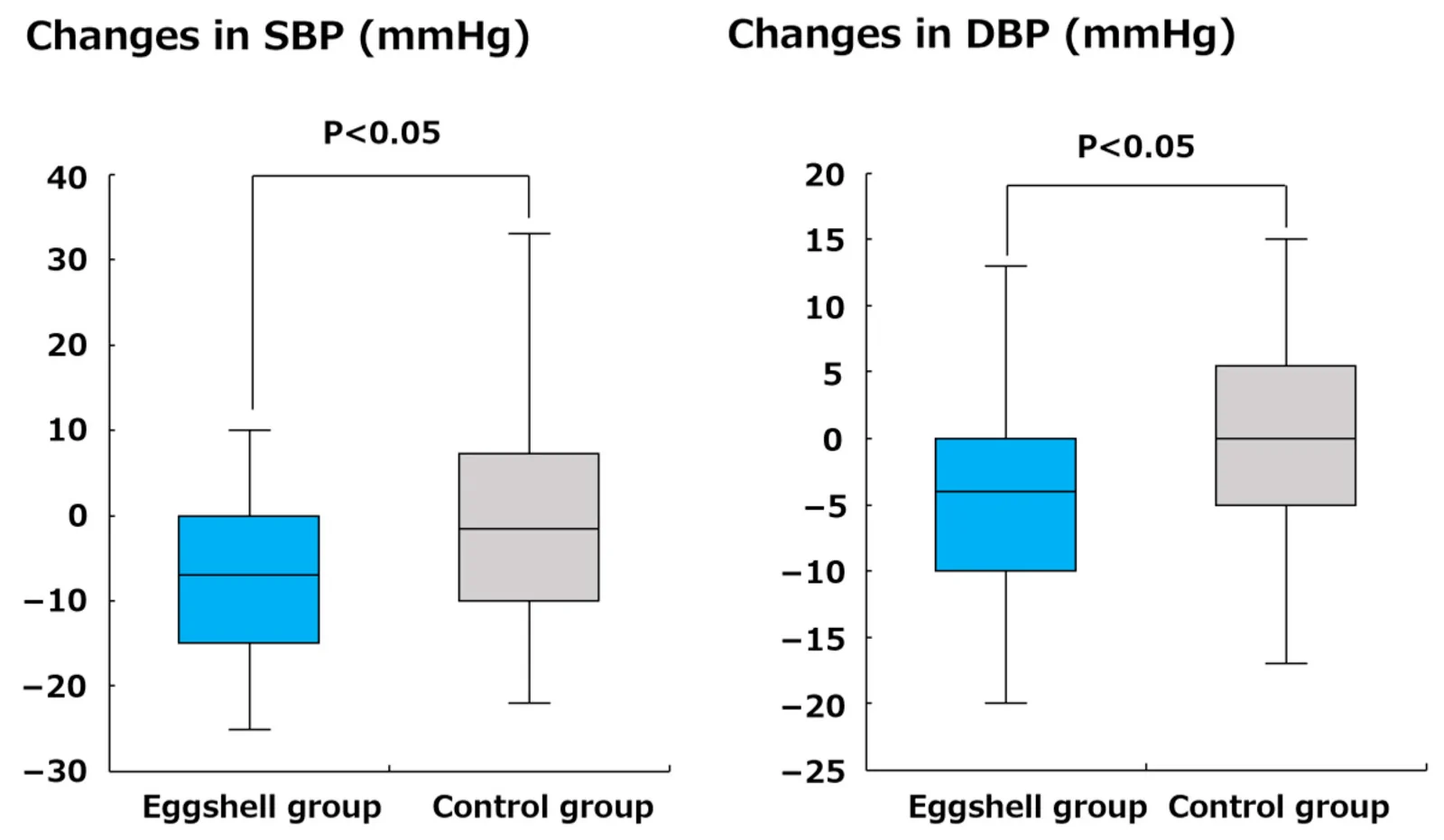
Boxplots comparing changes in blood pressure over 6 months [45].

The study included 94 women (mean age, 65 years) at high risk of hypertension living in rural communities on the outskirts of Hanoi, Vietnam. Subjects matched in pairs according to age, body mass index, and calcium intake were randomly assigned to either the control group or the eggshell calcium group, with 47 subjects in each group (five participants from each group dropped out for personal reasons). Two types of capsules were prepared for the intervention. One contained 100 mg of eggshell calcium (eggshell calcium group), and the other contained 100 mg of cellulose (control group). To provide equivalent nutritional support to the control group, identical amounts of thiamine and riboflavin, which do not affect calcium absorption or metabolism, were added to both capsules. Subjects consumed six capsules daily for six months. In the eggshell calcium group, systolic blood pressure (SBP) significantly decreased from 127 to 119 mmHg, while diastolic blood pressure (DBP) significantly decreased from 79 to 75 mmHg. However, no significant changes were observed in the control group. Furthermore, the reductions in both SBP and DBP were significantly greater in the eggshell calcium group than in the control group. These findings indicate that supplementation with 600 mg/day of eggshell calcium effectively lowers both SBP and DBP in elderly Vietnamese women at high risk of hypertension (Figure 7) [45].

One possible mechanism of this effect is that calcium deficiency stimulates parathyroid hormone secretion [41]. Parathyroid hormone promotes the release of calcium from bone into the bloodstream. The resulting increase in circulating calcium promotes calcium influx into vascular smooth muscle cells, causing vasoconstriction and thereby increasing blood pressure [46]. These findings suggest that eggshell calcium lowers blood pressure by decreasing circulating parathyroid hormone concentrations, thereby reducing calcium influx into vascular smooth muscle cells.

## 7. Inhibition of Fat Absorption by Eggshell Calcium

The effect of eggshell calcium intake on fat absorption was evaluated in nine healthy subjects in a crossover study by administering chocolate containing eggshell calcium or control chocolate and measuring fecal fatty acid levels. Fat excretion was significantly higher after consumption of chocolate containing eggshell calcium than after consumption of control chocolate [47]. Saturated fatty acids are generally reported to be poorly absorbed [48,49], and this study also showed increased excretion of saturated fatty acids. A possible mechanism is that fatty acids hydrolyzed by lipase in the gastrointestinal tract bind to calcium and become insoluble, thereby suppressing fatty acid absorption [50]. Eggshell calcium is more readily dissolved and ionized by gastric acid than calcium carbonate. Therefore, it was speculated that eggshell calcium may bind more readily to fatty acids in the intestinal tract than conventional inorganic calcium salts. The same mechanism may have contributed to the findings of this study. However, because eggshell calcium itself is highly absorbable, further studies are needed to clarify the underlying mechanisms of this phenomenon. Furthermore, the small number of subjects was a limitation of the study cited as Reference No. 47; it was considered necessary to conduct a re-examination with a larger sample size to clarify the effects.

## 8. Absorbability and Health Functions of Eggshell Membrane

The eggshell membrane is a protein-rich thin layer inside the eggshell. It has been used as an adsorbent for carbon dioxide and heavy metals [51,52] and in soy sauce development [53]. The main component of the eggshell membrane is protein, and its amino acid composition is shown in Table 2 [54]. Although eggshell membrane has been reported in human studies to exert effects such as skin moisturizing activity and improvement of knee joint pain [55,56,57,58], it remains unclear whether eggshell membrane protein is absorbed in the body. Therefore, we evaluated the absorption rate and net protein utilization following ingestion of eggshell membrane protein. In addition, the enzymatic hydrolysate of eggshell membrane protein was also evaluated.

Twenty-four male SD rats (245–275 g) were divided into four groups and pair-fed for 10 days with an AIN-76-based diet containing casein, eggshell membrane protein, or eggshell membrane enzymatic hydrolysate, or a protein-free diet for the measurement of metabolic nitrogen excretion. Feces and urine were collected from day 5 of feeding, and their nitrogen contents were measured to calculate net protein utilization.

As a result, the absorption rates of eggshell membrane protein and eggshell membrane hydrolysate were 94.8% and 87.0%, respectively, both of which were significantly lower than that of casein (98.5%). Conversely, net protein utilization was 84.6% and 84.7%, a significantly higher value than the 75.1% for casein. In addition, although the absorption rate showed a significantly higher value with ingestion of eggshell membrane hydrolysate than with intact eggshell membrane protein, no significant difference was observed in net protein utilization between these two protein preparations [54]. These results indicate that more than 80% of eggshell membrane protein is absorbed and utilized in the body, suggesting that it may contribute to its biological functions.

## 9. Discussion

This review summarizes the effects of eggshell calcium on bone metabolism, with a summary presented in Table 3. Calcium is one of the essential nutrients, is well recognized as an important nutrient for bone health, and has reference values, such as the Adequate Intake, established for calcium in the Dietary Reference Intakes in many countries. Globally, the WHO and FAO calcium intake standard for adult men and women is 1000 mg per day [59]. In addition, in Japan, calcium is classified as a nutrient in Foods with Nutrient Function Claims, and if a food provides 204–600 mg of calcium per daily intake, the claim “Calcium is a nutrient necessary for the formation of bones and teeth” may be displayed [60]. Calcium is also designated for a disease risk reduction claim under Foods for Specified Health Uses [61]. If the amount of calcium contained in the daily intake of a food is within the range of 300–700 mg and an application is submitted to and approved by the Consumer Affairs Agency, “This food is rich in calcium. Regular exercise and a healthy diet containing an adequate amount of calcium may help young women maintain healthy bones and reduce their risk of osteoporosis later in life.” may be displayed. Since the calcium intake of Japanese people is low compared to the dietary reference intake [30,62], an efficient and easily consumed source of calcium is needed to maintain bone health. As shown in this review, eggshell calcium has a porous structure, resulting in a large surface area in contact with gastric fluid after ingestion. Therefore, it is easily dissolved in gastric acid and is a highly absorbable calcium source. Since a larger contact area between calcium and stomach acid leads to better absorption, products made from finely pulverized eggshell calcium with reduced particle sizes are also available.

It has also been confirmed that the intake of eggshell calcium increases bone density in postmenopausal women. In addition to its effects on bone health, eggshell calcium has also been shown to lower blood pressure. This effect may be attributed to the suppression of bone resorption by eggshell calcium, which reduces the release of calcium from bone into the bloodstream. Consequently, calcium influx into vascular smooth muscle cells is reduced, thereby suppressing vascular contraction and contributing to blood pressure reduction.

As a recent topic, toothpaste containing eggshell-derived materials has been reported to promote tooth calcification, suggesting that eggshell components may have beneficial effects not only on bone but also on dental health [63,64].

Phosphorus is considered a nutrient that is difficult to eliminate in patients undergoing dialysis [65]. Administration of calcium to dialysis patients has been reported to reduce serum phosphorus concentrations [66]. Similar effects have also been reported with eggshell calcium supplementation [67]. The underlying mechanism is thought to involve the binding of eggshell calcium with phosphorus, resulting in its excretion from the body [68].

One of the challenges associated with calcium intake is that it may be difficult to obtain the required amount through ordinary foods alone. Milk and dairy products are well-known sources of calcium, but a relatively large amount of these foods may be required to achieve the recommended intake level. Vegetables are also considered calcium-rich foods, but some vegetables contain oxalic acid, which inhibits calcium absorption [69], making them a less efficient calcium source. Recently, methods to improve calcium absorption from vegetables have also been investigated [70]. In contrast, because eggshells are composed mainly of calcium carbonate, they contain a high amount of calcium in a small quantity. However, direct consumption of eggshells or eggshell powder is difficult. Therefore, incorporating eggshell calcium into supplements, calcium-fortified foods, or meal preparations may provide a practical approach for increasing calcium intake. Accordingly, the development of processed foods containing eggshell calcium is expected. In Japan, eggshell calcium products that enable increased calcium intake when added during rice cooking are commercially available. Eggshells have been reported to enhance the calcium content of various foods without adversely affecting taste [71]. In addition, the use of eggshell calcium has also been shown to improve the physical properties of certain food products [72,73]. Leveraging these characteristics, it is applied to bread, noodles, dairy products, processed meat products, and fruit juice or jelly beverages [74,75].

Furthermore, advancing the use of eggshell calcium would require an assessment from the perspectives of both economic viability and sustainability—considering factors such as the costs associated with raw material collection and processing, the feasibility of stable supply, and the environmental impact of the manufacturing process.

However, the availability of calcium-containing products, such as supplements, alone may not necessarily lead to sustained intake or improvements in nutritional status. Since nutritional education regarding calcium intake has been reported to increase calcium consumption [76], both access to calcium-containing foods and appropriate nutritional information are considered necessary to effectively increase calcium intake and promote health. It is crucial to expand strategies to bridge this gap.

Due to advances in medical care, the gap between healthy life expectancy and average life expectancy has increased, resulting in an extended period during which individuals may experience some form of disability, estimated to be approximately 10 years [77]. Metabolic syndrome, frailty, and dementia are considered major risk factors contributing to this burden [78]. The blood pressure-lowering effects by eggshell calcium may contribute to the prevention of metabolic syndrome, whereas its ability to increase bone mineral density may help prevent physical frailty, one of the major components of frailty. Furthermore, fractures are a major risk factor for becoming bedridden and may also indirectly increase the risk of dementia. The human studies involving eggshell calcium summarized in this review were conducted in Japan and Vietnam; while it was hypothesized—based on the mechanisms of digestion and absorption—that similar results would be obtained in other regions, it was concluded that verification is necessary.

## 10. Conclusions

In this review, we focused on eggshell calcium, an underutilized component of chicken eggs. As egg consumption increases, large amounts of eggshell waste are generated, making effective utilization of eggshells an important challenge. We hope that the findings presented in this review will contribute, even modestly, to the effective utilization of eggshells and the promotion of human health.

## Figures and Tables

**Table 1:** Nutritional content of eggshell calcium.

Nutrients	Contents (/100 g)
Moisture	0.3 g
Protein	2.1 g
Ash	96.8 g
Calcium	37.9 g
Potassium	36 mg
Sodium	76 mg
Magnesium	330 mg
Phosphate	93 mg

**Table 2:** Amino acid composition of eggshell membrane.

Amino Acids	Contents (g/100 g Dry Weight)
Ile	3.22
Leu	4.54
Lys	3.26
Met	3.71
Cys	9.73
Phe	1.54
Tyr	1.71
Thr	5.50
Trp	3.27
Val	6.92
His	3.95
Arg	7.13
Ala	2.59
Asp	8.19
Glu	12.4
Gly	5.84
Pro	9.36
Ser	5.21

**Table 3:** Summary of the health functions of eggshell calcium.

Organ/Tissue	Parameter	Human/Animal	Change
Stomach	Solubility in gastric acid	human	↑
Intestine	Apparent absorption rate	animal	↑
Serum	Parathyroid hormone	human	↓
Urine	Deoxypyridinoline	human	↓(N.S)
	Calcium excretion	animal	↓
Feces	Calcium excretion	animal	↓
	Fat excretion	human	↑
Bone	Bone mineral density	animal	↑
	SOS in bone	human	↑
Blood vessels	Blood pressure	human	↓

↑: Increase; ↓: Decrease; NS: not significant.

## Data Availability

No new data were created or analyzed in this study. Data sharing is not applicable to this article.

## Abbreviations

DBP	diastolic blood pressure
DPD	deoxypyridinoline
ELISA	enzyme-linked immunosorbent assay
PCA	passive cutaneous anaphylaxis
PTH	parathyroid hormone
QOL	quality of life
SBP	systolic blood pressure
SOS	speed of sound
